# Bronchiolitis obliterans organizing pneumonia (BOOP) after thoracic radiotherapy for breast carcinoma

**DOI:** 10.1186/1748-717X-2-2

**Published:** 2007-01-03

**Authors:** Robin Cornelissen, Suresh Senan, Imogeen E Antonisse, Hauw Liem, Youke KY Tan, Arjan Rudolphus, Joachim GJV Aerts

**Affiliations:** 1 Dept of Pulmonary Diseases, Sint Franciscus Gasthuis, Rotterdam, The Netherlands; 2 Dept of Pulmonary Diseases, Erasmus Medical Center, Rotterdam, The Netherlands; 3 Dept of Radiation Oncology, VU University Medical Center, Amsterdam, The Netherlands; 4 Dept of Radiation Oncology, Erasmus Medical Center, Rotterdam, The Netherlands; 5 Dept of Pulmonary Diseases, Lievensberg Hospital, Bergen op Zoom, The Netherlands

## Abstract

Common complications of thoracic radiotherapy include esophagitis and radiation pneumonitis. However, it is important to be aware of uncommon post-radiotherapy complications such as bronchiolitis obliterans organizing pneumonia (BOOP). We report on two patients with carcinoma of the breast who developed an interstitial lung disease consistent with BOOP. BOOP responds to treatment with corticosteroids and the prognosis is generally good despite of the need for long-term administration of corticosteroids as relapses can occur during tapering of steroids. This report provides guidelines for the evaluation and treatment of patients with pulmonary infiltrates after radiotherapy.

## Background

Radiation pneumonitis and fibrosis are well-recognized complications of thoracic radiotherapy, but less common complications include Bronchiolitis Obliterans Organizing Pneumonia (BOOP) and eosinophilic pneumonia [1]. It is also not commonly appreciated that these complications can manifest in patients receiving radiotherapy for breast cancer. We report two such patients who developed a BOOP following post-operative radiotherapy to the thoracic wall. The clinical features, diagnostic considerations, and treatment of interstitial lung disease following radiotherapy will serve to alert clinicians to this clinical entity and provide guidelines for diagnostic workup.

## Case report

### Patient no.1

A 59-year-old female who was a lifelong non-smoker underwent a modified radical mastectomy in August 2002 for an adenocarcinoma of her left breast, staged pT2N2M0. Adjuvant chemotherapy consisting of 4 cycles of doxorubicin (60 mg/m^2^) with cyclofosfamide (600 mg/m^2^) was administered from September to November 2002, followed by tamoxifen 20 mg daily. The patient was then referred for adjuvant radiotherapy on the left thoracic wall and the axillary lymph nodes. After CT planning, she received radiation from 6^th ^February to 20^th ^March 2003 to a total dose of 50 Gy in 25 fractions. The thoracic wall was irradiated using tangential 6 Mv photon fields, and regional lymph nodes using an anterior-posterior photon field (6 Mv) dosed at 3 cm, with a posterior top-up field to the axilla. The V_20_, i.e. volume of total lung receiving a dose of 20 Gy or more, was 32%.

In April 2003, the patient complained of shortness of breath. Physical examination revealed a temperature of 38.0°C and pulmonary auscultation revealed inspiratory crackles and bronchial breathing sounds. Peripheral O_2 _saturation was 97% measured by a pulse-oxymeter during treatment with 2 litres of O_2 _a minute. Blood analysis revealed a CRP of 189 mg/L(0–10 mg/L) and a one-hour sedimentation rate of 105 mm/hour(0–30 mm/hour), haemoglobin was 5.4 mmol/L(7.5–10.0 mmol/L), WBC count was 6.0 × 10^9^/L(4.4–10.0 × 10^9^/L) with a slight eosinophilia of 12%(0–5%), platelet count was 365 × 10^9^/L(150–400 × 10^9^/L). Further serum chemistry, renal, liver functions, and urinalysis were normal. A chest radiograph showed a patchy consolidation zone in the upper lobe and the apex of the lower lobe of the left lung. Computer tomography (CT) revealed multiple ground glass opacities in the upper and lower lobe of the left lung, and no abnormalities were seen in the right lung.

**Figure 1:**
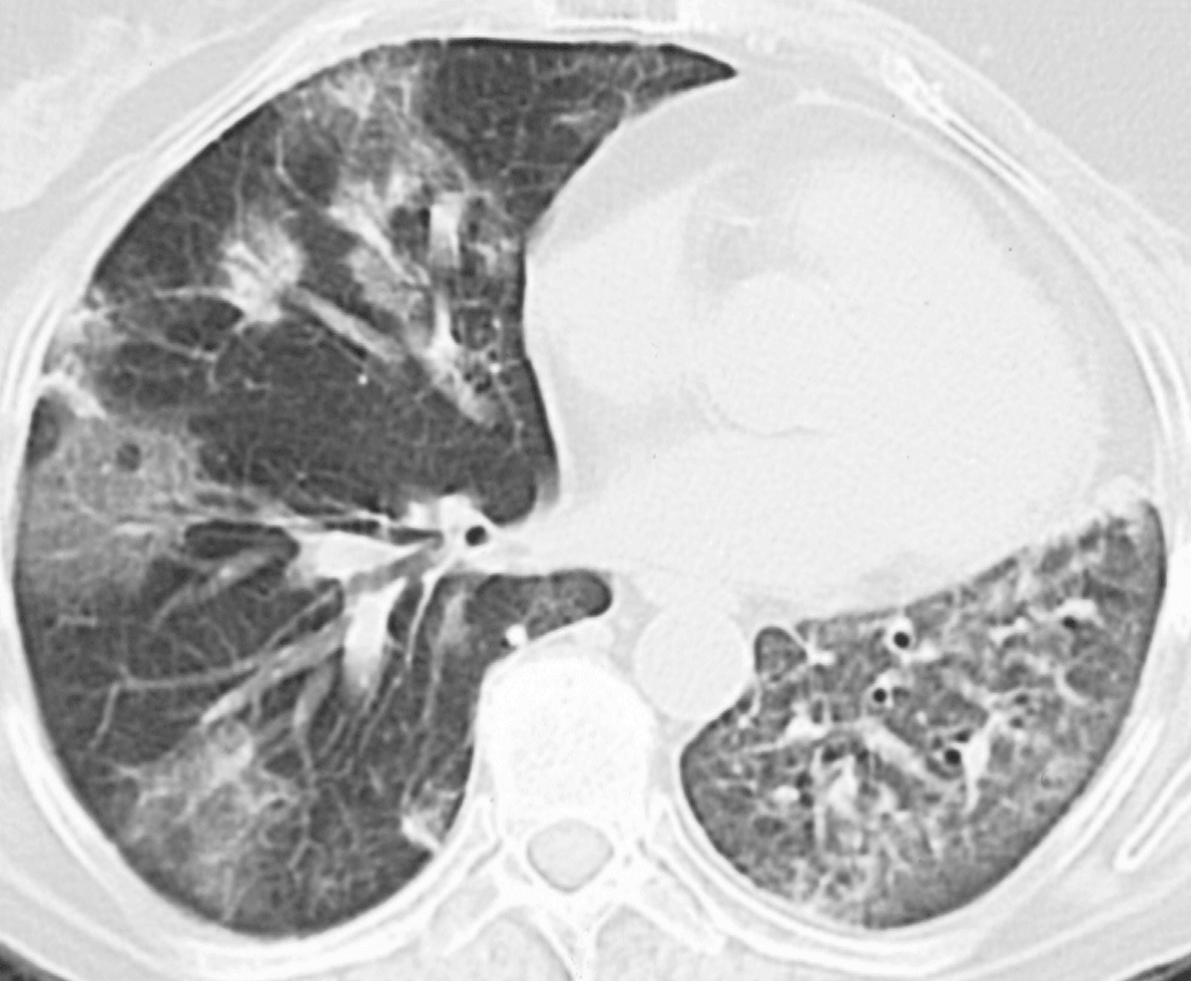
CT shows patchy areas in the left lung and patchy areas in the middle lobe of the right lung.

**Figure 2:**
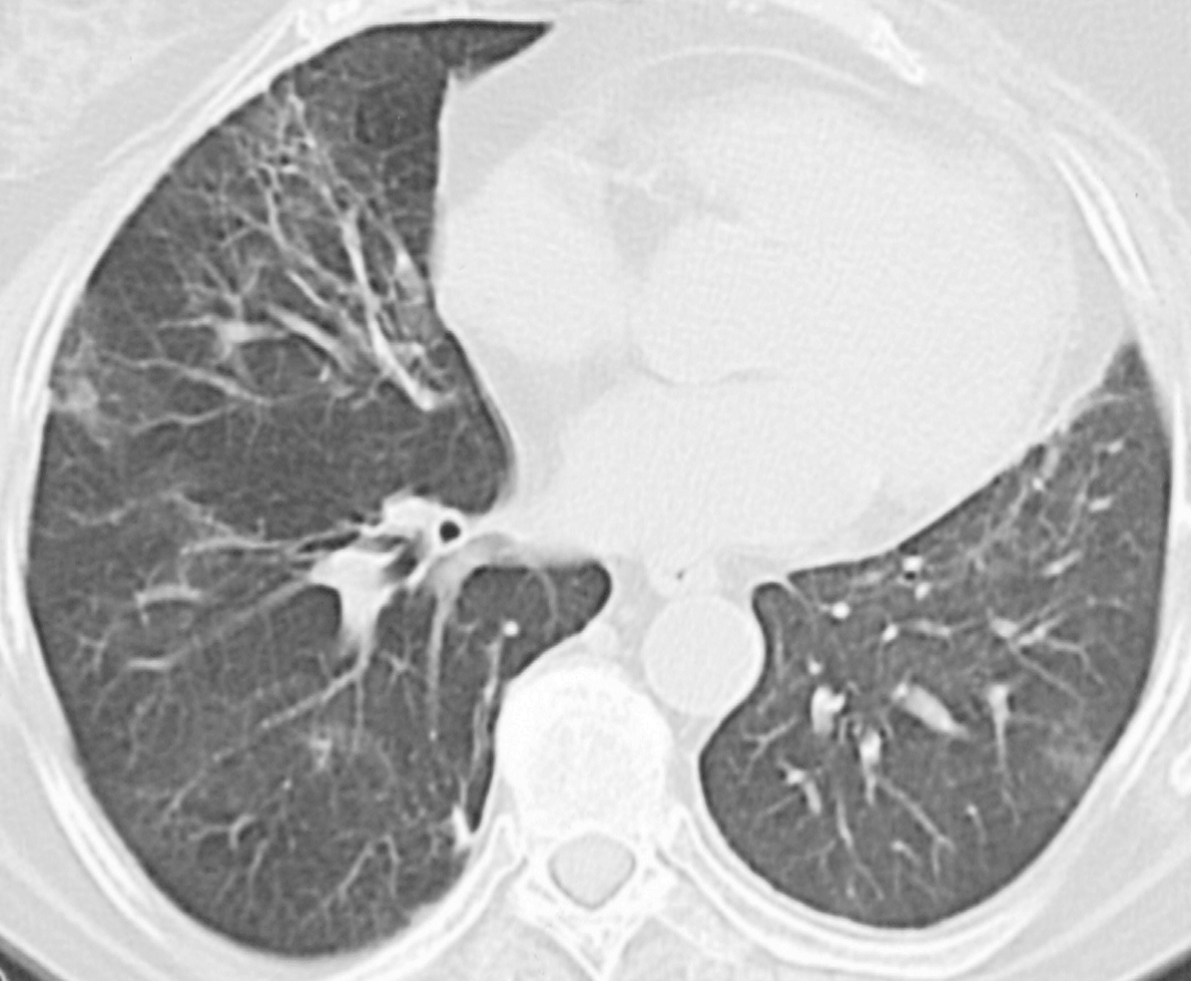
CT shows an obvious improvement of the patchy infiltrates in the right lung after reintroduction of steroids.

A clinical diagnosis of radiation pneumonitis was made and treatment with prednisone 20 mg 3 times daily was initiated, with an improvement in clinical symptoms seen within 4 days. In the following months, the dose of prednisone was gradually tapered to 15 mg once daily. The chest radiograph in July 2003 showed a reduction of the ground glass opacities. In August 2003, patient was hospitalised due to shortness of breath. A new CT showed an increase of the patchy infiltrates in the left lung, but also new patchy infiltrates in the middle lobe of the right lung (figure 1). To rule out an infectious cause, a bronchoscopy was performed which revealed no endobronchial abnormalities. No biopsies were performed but broncho-alveolair lavage showed a normal cell count and negative cultures. Consequently, a diagnosis of interstitial lung disease due to radiotherapy was made, most probably BOOP, and the prednisone dosage was increased to 20 mg four times daily. A clinical improvement was seen within two weeks, after which the prednisone dosage was tapered in the following 12 months. Radiological improvement was observed in the right lung (figure 2). However, abnormalities consistent with radiation fibrosis remained within the radiation field in the left lung.

### Patient no.2

A 92-year-old patient presented with multiple intradermal metastases from a breast cancer on the right thoracic wall 10 years after undergoing an amputation of the right breast for a pT3N0M0 breast cancer. No adjuvant post-surgical therapy was administered after the initial surgery. Restaging revealed no regional or distant metastasis and the chest wall lesions were resected. Pathological examination showed an irradical resection after which she was referred for radiotherapy. She received thoracic and axillary radiotherapy to a total dose of 60 Gy in once-daily fractions of 2 Gy using non-CT based planning. Radiotherapy to 50 Gy was performed using an anterior electron field (10 MeV) matched to a lateral photon field (6 Mv) dosed at 3 cm. The dose on the axilla was supplemented using a posterior field. This was followed by a boost of 10 Gy to the site of irradical excision on the chest wall using 10 MeV electrons.

Three months after completing radiation therapy, the patient was hospitalised for complaints of fever and shortness of breath. She was febrile (38.0°C) and auscultation revealed bronchial breath sounds over the right lung while no abnormalities were heard on the left side. The one-hour sedimentation rate was 98 mm/hour(0–30 mm/hour), WBC count was 9.8 × 10^9^/L(4.4–10.0 × 10^9^/L). Chest radiograph showed a density of the right upper lobe and, to a lesser degree, the right lower lobe of the lung. The left lung showed a normal pattern. Bronchoscopy revealed no endobronchial abnormalities or evidence for infection. Bronchial biopsies showed normal tissue.

A diagnosis of radiation pneumonitis was made and high-dose prednisone 40 mg once daily was started. The clinical condition of patient improved significantly within two weeks and patient was discharged. During follow up, the improved clinical condition and radiological imaging lead to a tapering of the prednisone, with discontinuation after 6 months. The symptoms recurred two months later and a chest radiograph showed an increase in the density of the right lung and a density in the left upper lobe. As the radiological abnormalities were migrating beyond the radiation field, a diagnosis of BOOP following radiotherapy was made. Treatment with prednisone 10 mg was resumed and led to a resolution of her symptoms within two weeks. The prednisone dose was gradually tapered and totally discontinued again after 6 months.

## Discussion

Despite the frequency of radiotherapy for breast cancer, the development of interstitial lung disease complicating this therapy is uncommon. A retrospective analysis in 451 patients found clinical symptoms and chest radiographs compatible with radiation pneumonitis in 5.5% of breast cancer patients [2]. Next to radiation pneumonitis, the commonest interstitial lung diseases after radiotherapy are BOOP and chronic eosinophilic pneumonia [1]. BOOP is a rare pulmonary disorder, which has wide range of causes, such as infection, inhalation of toxic agents and medication. BOOP is associated with the presence of intraluminal plugs of connective tissue in bronchioles extending to the alveoli, with a patchy distribution and a preservation of the background architecture [3]. The pathophysiology of BOOP after radiotherapy of the chest is unknown.

The first radiographic change is usually a diffuse haze in the irradiated lung, which progresses to patchy alveolar infiltrates with air bronchograms [4]. A migratory pattern of dense alveolar infiltrates in both radiated and non-irradiated lung zones can be seen [5-8]. Patients who have received unilateral thoracic radiotherapy show activity on a ^18^fluoro-2-deoxyglucose (FDG) positron emission tomography (PET) in the ipsilateral and contralateral lung [9]. Common laboratory findings include polymorphonuclear leukocytosis and an elevated erythrocyte sedimentation rate. This sedimentation rate can be very high, up to 140 mm/h [7,8]. A bronchio-alveolar lavage (BAL) often reveals a lymphocytosis, and both neutrophilia and eosinophilia can also be found [7]. Open lung biopsy is the preferred method for establishing the diagnosis [10], however transbronchial biopsy has also been used [3].

From a radiological and clinical point of view, both BOOP and radiation pneumonitis may have a similar presentation. Radiation pneumonitis is also associated with an interstitial pulmonary inflammation with also an alveolar exudative component [11]. However, radiation pneumonitis can lead to irreversible lung damage and evolve to radiation fibrosis. BOOP tends to arise several months after the completion of radiotherapy which is in general longer than radiation pneumonitis [12]. While radiation pneumonitis is generally limited to the irradiated fields, migration of alveolar opacities is characteristic of BOOP [13,14]. Both our patients were treated for a radiation pneumonitis before the BOOP was diagnosed, and while it is not possible to exclude BOOP as the initial presentation, the initial symptoms were more compatible with a radiation pneumonitis [15]. The mechanisms of BOOP co-existing with radiation pneumonitis are unknown [16], but both were present in both our patients.

Unilateral irradiation for breast carcinoma has been reported to induce an increase of lymphocytes with elevated CD4/CD8 ratio in broncho-alveolar lavage fluid in both the contralateral and ipsilateral lung soon after radiotherapy [17]. These infiltrates would spontaneously disappear in most patients but might gradually consolidate in a small number of patients and manifest as BOOP [12]. Some reports suggest that radiation pneumonitis is linked to the combination of tamoxifen and radiotherapy [12], but no such correlation was found for BOOP [18,19].

Apart from BOOP, chronic eosinophilic pneumonia has also been described in patients after radiotherapy, and almost exclusively in patients with a history of asthma or allergy [1]. Clinical symptoms arising from this interstitial lung disease are similar to those of BOOP, but blood and BAL cell count do show a more pronounced eosinophilic inflammation. The differential diagnosis of an interstitial lung disease complicating radiation therapy includes radiation pneumonitis, lymphangitis carcinomatosa and infections such as tuberculosis, all of which can have major clinical consequences for the patient [1,3,5-8,12,14]. Due to a lack of awareness of the diagnosis, invasive investigations like video assisted thoracoscopy (VATS) have been performed in order to obtain a diagnosis. A bronchoscopy can exclude the other diagnoses [13].

We propose that a clinician should be aware of the possibility of a BOOP in a post-radiotherapy setting, and after exclusion of an infectious cause by bronchoscopy, is justified in starting treatment with corticosteroids. All reported interstitial lung diseases in patients after radiotherapy irrespective of the precise diagnosis are described to respond dramatically well to steroids [16]. In the largest patient series described for BOOP, *Epler *recommended 1 mg/kg for 1 to 3 months, then 40 mg/d for 3 months, then 10 to 20 mg/d or every other day for a total of 1 year. Treatment shows spectacular improvement, clinically in one week and radiological resolution usually follows in between 2 to 4 weeks [16]. Short-term therapy leads to recurrences [7,8], which appear between one to six weeks of discontinuation or tapering of the steroid treatment. The long-term outcome of interstitial lung diseases after radiotherapy appears excellent, when treatment with corticosteroids is initiated promptly [16]. In our second patient a reduced dose of steroids was chosen because of the high age of the patient.

Although not unique, we think that our case reports serve to increase the awareness of clinicians to include interstitial lung disease in the differential diagnosis of patients presenting with lung infiltrates after radiotherapy. Additional investigations can be reserved for patients in whom quick resolution of infiltrates is not observed.
